# Notes on Preparations for the Sick

**Published:** 1908-06

**Authors:** 


### Notes on Preparations for the Sick.

**Novaspirin.**—The Bayer Co., London.—Novaspirin passes unchanged through the stomach, and therefore is not likely to cause gastric disturbances. In the intestines it decomposes into nascent salicylic acid and methylene-citric acid, without giving rise to irritation or unpleasant by-effects, such as tinnitus aurium, headache, cardiac depression, or renal irritation.

Chemically, Novaspirin is methylene-cytril-salicylic acid. It is a white, odourless powder of faint acidulous taste, easily soluble in alcohol, difficultly soluble in chloroform and ether, and almost insoluble in water. It contains 62 per cent. of salicylic acid.

Novaspirin has proved an excellent remedy in influenza, in which it promptly relieves the distressing pains, reduces the fever, and in general promotes the comfort of the patient. It is also equally valuable in the treatment of colds, such as coryza, tonsillitis and pleurisy, neuralgia, nervous headaches, tabes and carcinoma. The administration of Novaspirin is not followed by profuse perspiration, because the drug is absorbed so slowly—a matter of importance when diaphoresis is not desired, as in cases of weak patients, grave cardiac affections, chronic wasting diseases, and danger of severe hemorrhages—nor has ringing in the ears been observed to follow the use of the drug. It is an efficient and agreeable remedy in the various forms of rheumatism, both articular and muscular, as well as in gouty conditions, and is also highly recommended in neuralgias—especially sciatica and intercostal neuralgia—and other painful affections.

The average dose for adults is 15 grs. several times a day; this may be increased to 30 grs. in rheumatic and gouty troubles. Owing to its insolubility in water, it is best administered in powders, wafers, or in the form of tablets.

---

**Lecithin** (Fairchild).—BURROUGHS, WELLCOME & CO., London.—Fairchild's various preparations have gained the esteem of medical men by their excellence, so that any comment upon them is quite unnecessary.

The product mentioned above is a liquid, containing one grain of lecithin in each teaspoonful. It is a very convenient way of administering this organic phosphorus compound, and its general properties render it an "elegant" production of modern pharmacy.

---

**Kepler Malt Extract.**—BURROUGHS, WELLCOME & CO., London.—This is now packed in a new bottle of much improved design, fitted with a cork-lined metal lid. The neck of the bottle is large enough to admit a dessert-spoon, and a cork-lined metal cap takes the place of the cork. This cap closes the bottle efficiently, and it is secured by a metal band which clips the cap and the rim of the mouth of the bottle. The whole arrangement is much more convenient and cleanly than anything previously designed.

---

**Hemisine.**—BURROUGHS, WELLCOME & CO., London.—The active principle of the medulla of the suprarenal gland. This solution of the crystalline active principles, physiologically standardised to one in a thousand, may be used as it is or diluted with normal saline, obtained by dissolving soloids of sodium chloride. Locally, it produces an intense anæmia of the mucous membrane. It may be prescribed internally: (1) To produce a local vaso-constrictor effect on the mucous membrane of the alimentary canal, especially of the stomach, *e.g.* to arrest hemorrhage from acute gastric ulcer, 5 minims of Hemisine 1 in 1,000 solution, in water, or one "Tabloid" Hemisine, 0.0003 gramme (approximately gr. 1/200), are administered, and repeated as required. (2) To exert, after absorption, a tonic action on the sympathetic nervous system, on the circulation, and on the uterus. For this purpose 15 minims of Hemisine 1 in 1,000 solution, in water, or one "Tabloid" Hemisine, 0.001 gramme (approximately gr. 1/64), are given two or three times daily, according to the reaction of the patient.

Hemisine products are issued in a variety of forms for various purposes, and in various compounds with eucaine, cocaine, atropine, etc. The wide range of utility of this large series of preparations is obvious.

---

**"Tabloid" Phenol and Menthol Compound.**—Burroughs, Wellcome & Co., London.—Each contains :—

Phenol, gr. $\frac{1}{4}$.

Menthol, gr. $\frac{1}{2}$.

Oil of Cajuput, min. 1.

"Tabloid" Phenol and Menthol Compound will be found a pleasant and effective means of administering this gastric stimulant and antiseptic. The combination is useful to promote appetite and digestion, to relieve pain in dyspepsia, and to prevent or arrest fermentation in dilated stomach. It is of service in flatulence, fermentative dyspepsia, and in those cases where stomach lavage is commonly practised. It exercises an antiseptic and carminative effect in the intestine, and so is of value in griping, and in cases where improper fermentative changes occur in the bowel. The gelatine envelope preserves the contents from the action of the atmosphere, and the shape facilitates swallowing.

---

**Analgesic Balm.**—Parke, Davis & Co., London.—This preparation is a combination of methyl salicylate, menthol and lanoline. It forms an efficient means of applying these remedies in the treatment of arthritic inflammations and painful affections generally. It is very effectual in nerve pains, in trigeminal and intercostal neuralgias, sciatica, etc., and applied to the forehead in frontal headache or migraine, it affords relief from pain. It also forms a very useful dressing for small contusions and for sprains, provided the skin is not broken. The collapsible tubes in which it is packed are very convenient for storage or transport, and for expelling the necessary quantity for the occasion, whilst the bulk is in no way exposed to deterioration.

---

**Pill Aloin and Phenolphthalein Compound.**—Parke, Davis & Co., London.—This combination of aloin, strychnine, belladonna, phenolphthalein and ipecacuanha, has been found very effective in the treatment of chronic constipation, and for the relief of migraine, hepatic torpor, and kindred disorders. The ingredients are carefully assayed to ensure reliable activity, and the pill operates promptly and thoroughly, yet without discomfort, whilst its gelatine coating effectually conceals the taste of the medicaments.

---

**Solurol: Thyminic Acid, a new remedy for gout.**—ALLEN & HANBURY, London.—This drug is described as the natural organic solvent of uric acid, and has been given with much success in those gouty and rheumatic conditions which are due to an excess of uric acid. In such cases the painful symptoms of the disease are quickly alleviated, and the duration of an attack is very much shortened. Sometimes in an acute attack of gout the pain and inflammation may be somewhat increased for a short period, but this quickly passes. If Solurol be taken regularly on the first threatening of an attack of gout, the attack can often be aborted.

It has no prejudicial or depressing action, but simply helps the elimination of uric acid by retaining it in solution, thus preventing the deposition of urates in the tissues.

A four-grain tablet may be taken three times a day.

---

**Fermenlactyl.**—ANGLO-AMERICAN PHARMACEUTICAL CO., Croydon.—A pure lactic ferment, prepared from the special bacillus of Bulgarian Milk from cultures isolated in the Pasteur Vaccine Company's laboratories of Paris. It is put up in tablet form. These may be crunched and swallowed, or may be used for the preparation of a special Bulgarian Milk diet in accordance with the special directions given.

Fermenlactyl is indicated in the following diseases :—

(*a*) Affections of the digestive tube, gastro-enteritis, infantile diarrhœa, dysentry, intestinal tuberculosis, typhoid fever, chronic intestinal infection, and for the treatment of diabetes, in which disease the lactic ferment destroys the sugar.

(*b*) Fermenlactyl is also indicated for reducing to a minimum the source of organic intoxication, cirrhosis of the liver, chronic nephritis, cardiac affections, gout, gravel, arterio-sclerosis (old age), arthritis, furunculosis, urticaria.

(*c*) Fermenlactyl may also be prescribed in cases where patients are submitted to an alimentary diet liable to increase intestinal fermentation, such as the feeding of children with sterilised and unsterilised milk.

Those who adopt the views of Professor Metchnikoff as to the causes and prevention of old age will doubtless be disposed to give this preparation a prolonged and persevering trial.

**Ovaltine.**—A. WANDER, 24 Queen Victoria Street, London.—Ovaltine is a food for children and invalids, its special feature being the presence of Lecithin.

As is well known to the medical profession, Lecithin is an organic phosphorus compound, which can be extracted from nervous tissues, and which acts as a nerve restorative.

Ovaltine has certainly one great advantage over many artificial foods in not being excessively sweet, but yet possessing a very pleasant taste.

A portion was ignited and the ash examined for the presence of phosphorus, which was found present to a marked extent. We have every confidence in recommending a trial of this food.

---

**Formitrol Pastilles.**—A. WANDER, Berne and London.—These pastilles contain formaldehyde, menthol, citric acid, and sugar of milk. They should have an excellent effect in favouring oral antisepsis in infectious conditions of the mouth and throat.

---

**"Ju-Vis" Preparations.**—FOSTER, CLARK & CO., Maidstone.—The preparations made by Messrs. Foster, Clark & Co. have been before the public for some considerable time, and are gaining for themselves a good reputation. The distinguishing feature is their low price, combined with satisfactory quality.

Ju-Vis is an extract of meat combined with vegetables, which can be obtained either as a liquid, or in the form of tablets

These extracts are accompanied by the makers' guarantee of purity, which is not always the case with similar substances.

We have examined a specimen of the tablets, and find them to consist of the ingredients stated; they are therefore valuable, and are also palatable food products.

---

**Armour's Pharmaceutical Preparations: Nutritive Elixir of Peptones, Glycerine Extract of Red Marrow.**—Holborn Viaduct, London, E.C.—The firm of Armour & Co. has very excellent facilities for producing medicinal preparations of animal origin, ranging from extract of beef to the more active principles obtained from various "ductless glands."

From time to time we have examined these products, but have recently paid special attention to the above-mentioned examples of the enterprise of Messrs. Armour & Co., namely Nutritive Elixir of Peptones, and Glycerine Extract of Red Marrow. The former contains no coagulable proteid, but gives reactions for peptones, and should therefore be readily assimilated. It contains a small amount of alcohol, which is probably derived from the sherry used in its manufacture. It may be well described as a "Nutrient Restorative."

The extract of Red Bone Marrow is a pleasantly flavoured elixir, and gives well-marked spectroscopic evidence of the presence of hæmoglobin. This renders it a valuable means of administering an organic compound of iron in cases of anæmia and similar diseases.

---

**Alexine.**—THOMAS CHRISTY & Co., London.—In the treatment of the fashionable complaint neurasthenia this granular powder is likely to become useful. It is composed for the most part of pure phosphoric acid, but in order to lend perfect stability to the preparation it is made to include a small portion of the bi-phosphate of manganese and a small quantity of the bi-phosphate of iron. It is readily soluble in water, to which it imparts an agreeable, slightly acid taste. The dosage is rendered particularly simple by the method in which the preparation is put up. The glass stopper is hollowed so as to contain an amount of the powder which, when dissolved in about three parts of a tumblerful of water, constitutes the dose for an adult. For children, between two and five years, this dose is halved. One great advantage of its agreeable taste is that it can easily be mixed with the beverages which are usually taken at meals, such as lemonade, wine, and the like.

Assimilable phosphorus is believed to be the food par excellence for the higher centres of the nervous system, hence a compound of this kind should be much in demand for cases of either real or fancied nervous debility, for which all the resources of the pharmacopœia are sometimes apparently useless. It should also materially shorten the prolonged convalesence after influenza and other acute diseases.

